# Development of a Magnetic Electrochemical Bar Code Array for Point Mutation Detection in the H5N1 Neuraminidase Gene

**DOI:** 10.3390/v5071719

**Published:** 2013-07-15

**Authors:** Ludmila Krejcova, David Hynek, Pavel Kopel, Miguel Angel Merlos Rodrigo, Vojtech Adam, Jaromir Hubalek, Petr Babula, Libuse Trnkova, Rene Kizek

**Affiliations:** 1 Department of Chemistry and Biochemistry, Faculty of Agronomy, Mendel University in Brno, Zemedelska 1, Brno CZ-613 00, Czech Republic; E-Mails: lidakrejcova@seznam.cz (L.K.); d.hynek@email.cz (D.H.); paulko@centrum.cz (P.K.); merlos19792003@hotmail.com (M.A.M.R.); vojtech.adam@mendelu.cz (V.A.); libuse@chemi.muni.cz (L.T.); 2 Central European Institute of Technology, Brno University of Technology, Technicka 3058/10, Brno CZ-616 00, Czech Republic; E-Mails: hubalek@feec.vutbr.cz (J.H.); petr_babula@email.cz (P.B.); 3 Department of Microelectronics, Faculty of Electrical Engineering and Communication, Brno University of Technology, Technicka 10, Brno CZ-616 00, Czech Republic; 4 Department of Natural Drugs, Faculty of Pharmacy, University of Veterinary and Pharmaceutical Sciences, Palackeho 1-3, Brno CZ-612 42, Czech Republic; 5 Department of Chemistry, Faculty of Science, Masaryk University, Kotlarska 2, Brno CZ-611 37, Czech Republic

**Keywords:** voltammetry, highly pathogenic influenza, antiviral resistance, paramagnetic particles, hybridization, quantum dots, automated separation, electrochemistry

## Abstract

Since its first official detection in the Guangdong province of China in 1996, the highly pathogenic avian influenza virus of H5N1 subtype (HPAI H5N1) has reportedly been the cause of outbreaks in birds in more than 60 countries, 24 of which were European. The main issue is still to develop effective antiviral drugs. In this case, single point mutation in the neuraminidase gene, which causes resistance to antiviral drug and is, therefore, subjected to many studies including ours, was observed. In this study, we developed magnetic electrochemical bar code array for detection of single point mutations (mismatches in up to four nucleotides) in H5N1 neuraminidase gene. Paramagnetic particles Dynabeads® with covalently bound oligo (dT)_25_ were used as a tool for isolation of complementary H5N1 chains (H5N1 Zhejin, China and Aichi). For detection of H5N1 chains, oligonucleotide chains of lengths of 12 (+5 adenine) or 28 (+5 adenine) bp labeled with quantum dots (CdS, ZnS and/or PbS) were used. Individual probes hybridized to target molecules specifically with efficiency higher than 60%. The obtained signals identified mutations present in the sequence. Suggested experimental procedure allows obtaining further information from the redox signals of nucleic acids. Moreover, the used biosensor exhibits sequence specificity and low limits of detection of subnanogram quantities of target nucleic acids.

## 1. Introduction

Highly pathogenic avian influenza A (HPAI), subtype H5N1, represents a threat to the human population [1]. While respiratory symptoms and fever are typical signs of influenza, H5N1 has a high incidence of neurological sequelae in many animal species and sporadically in humans, but it represents a continuous danger of global pandemic associated with high mortality [2,3]. HPAI viruses have caused millions of deaths in domestic poultry, and hundreds of deaths in humans [4]. Circulating of influenza viruses in wild animals poses the risk to human health [5,6]. Humans can be infected also by animal subtypes, such as avian influenza virus H5N1 and H9N2 and swine influenza virus H1N1 and H3N2 [7,8,9]. The primary risk factor for human infection appears to be direct or indirect exposure to infected live or dead animals or contaminated environments [10]. H5N1 occurs in two distinct pathotypes in bird population, seasonal low pathogenic avian influenza (LPAI), and highly pathogenic avian influenza (HPAI) [11,12,13,14]. LPAI may become HPAI to poultry through mutations after introduction from wild birds to poultry, but only two subtypes (H5 and H7) can become HPAI [15,16,17]. These viruses may result in 100% mortality within a susceptible poultry species [16]. The mechanism of mutation of LPAI to HPAI is based on passage in susceptible animals, typically poultry during several months. New generated HPAI virus has broken out in flocks of poultry as so as in wild birds, and caused devastation with huge economic and ecologic impact [3,18]. HPAI poses risk not only for birds, there were also reported sporadic human infections with low morbidity but high mortality, nearly 60% [19,20,21].

HPAI is not capable of droplet infection from human to human. For this reason, spread of H5N1 virus in the human population is limited [22]. There is a concern that H5N1 may obtain this ability and become pose a potential pandemic hazard to public health worldwide [23,24,25,26,27]. Influenza viruses can develop resistance to pharmacological mechanism of neuraminidase inhibitors (NAIs) that is based on the loss of binding affinity of these drugs. Antiviral resistance in influenza may not develop entirely during treatment but also sometimes transmit widely to replace susceptible strains in the absence of drug pressure [28]. Recently, different authors showed different methods for determination of mutations in H5N1. Sparse learning method was developed to identify antigenicity-associated sites in highly pathogenic H5N1 influenza virus HA based on immunologic data sets [29]. The analysis of complete genome sequences, genetic evolution and phylogenetic analyses showed that the sequence analysis of H5N1 influenza virus displayed the drug-resistant mutations in the matrix protein and NA genes [30]. This fact is important for better understanding the prevalence and adaptation of H5N1 influenza viruses in different countries [31,32]. The continuous mutations in NA gene give rise to a great necessity to monitor its sequence for detection of any possibilities to drug resistance in H5N1 [33,34,35,36].

The current study was targeted to detect possible mutations in NA gene for both diagnosis and treatment purposes. Neuraminidase inhibitors resistance is based on single-point mutations of neuraminidase gene (H 275 Y) [37,38]. Our choice of three various gene sequences for neuraminidase of H5N1 influenza was based on this assumption. There was chosen sections, in which the sequences differed from each other. This way was considered as a model of point mutation. Further, we report multi-target detection of point mutation in H5N1 NA gene. As a model real sample RNA oligonucleotide (RNA ODN) labeled with CdS was tested. Isolation and detection was carried out under conditions optimized by labeled DNA oligonucleotide. In addition, we described hybridization assay based on automatic isolation by modified paramagnetic particles (MPs). Isolated target molecule labeled by quantum dots (QDs) was detected by electrochemical analysis. 

## 2. Experimental Section

### 2.1. Chemicals

All used chemicals in ACS purity were purchased from Sigma Aldrich (Sigma-Aldrich, St. Louis, MO 3050 Spruce St., St. Louis, MO 63103, USA) unless noted otherwise. Stock solutions were prepared with ACS water. The pH value was measured using inoLab Level 3 (Wissenschaftlich-Technische Werkstatten GmbH; Weilheim, Germany). Deionized water underwent demineralization by reverse osmosis using Aqua Osmotic 02 (Aqua Osmotic, Tisnov, Czech Republic) and was subsequently purified using Millipore RG (MiliQ water, 18 MΏ, Millipore Corp., Billerica, MA 3050 Spruce St., St. Louis, MO 63103, USA). Deionized water was used for rinsing, washing and buffer preparation.

### 2.2. Preparation of QDs (CdS, PbS and ZnS)

All chemicals were purchased from Sigma-Aldrich (St. Louis, MO, USA) and used without further purification. CdS quantum dots (QDs) were prepared with a slightly modified method published in [39]. Briefly, cadmium nitrate tetrahydrate Cd(NO_3_)_2 _· 4H_2_O (0.0309 g, 0.1 mM) was dissolved in ACS water (25 mL). 3-mercaptopropionic acid (35 µL, 0.4 mM) was slowly added to stirred solution. Afterwards, pH was adjusted to 9.11 with 1 M ammonia solution (NH_4_OH, 1.5 mL). Sodium sulphide nanohydrate Na_2_S · 9H_2_O (0.0240 g, 0.1 mM) in 23 mL of ACS water was poured into the first solution with vigorous stirring. Obtained yellow solution was stirred for 1 h. ZnS QDs were prepared similarly to CdS QDs; zinc nitrate hexahydrate Zn(NO_3_)_2_·6H_2_O (0.0298 g, 0.1 mM) was used in the preparation. Colorless solution was obtained.

PbS QDs were prepared by modified method of Hennequin [40]. Lead acetate trihydrate Pb(OAc)_2_·3H_2_O (0.0379 g, 0.1 mM) was dissolved in ACS water (25 mL). 3-mercaptopropionic acid (60 µL, 0.69 mM) was slowly added to stirred solution. White precipitate, which disappeared after addition of 3.8 mL of 1 M ammonia solution (pH 9.88), was formed. Sodium sulfide nonahydrate Na_2_S · 9H_2_O (0.0120 g, 0.05 mM) in 21.2 mL of ACS water was added under vigorous stirring. Color of solution was brown. All QDs solutions were stored in dark at 4 °C prior to use.

### 2.3. Labeling of Influenzas’ Derived DNA and RNA Oligonucleotides with QDs (CdS, PbS and ZnS)

Probes were found in the GenBank database at NCBI ([41], Table 1, Table 2) and synthesized by Sigma‑Aldrich. The number for Zhejiang, China and Aichi ODN were DQ643810, EU263982, and AB684120, respectively. Briefly, ODN-SH (DNA or RNA) (100 µL, 100 µg.mL^−1^) was mixed with a solution of NPs (100 µL, 100 µg.mL^−1^). This mixture was shaken for 24 h at room temperature (Vortex Genie2, Scientific Industries, 70 Orville Drive, Bohemia, NY, USA). Subsequently, solution was dialyzed against 2000 ML of miliQ water (24 h, 4 °C) using a Millipore membrane filter 0.025 µm VSWP. During dialysis the sample was diluted to 800 µL. Diluted sample was concentrated to the final volume of 500 µL on a centrifuge filter device Amicon Ultra 3k (Millipore, Merck Millipore Headquarters 290 Concord Road, Billerica, MA 01821, USA). Centrifuge 5417R (Eppendorf, Hamburg, Germany) was set to the following parameters: 15 min, 4,500 rpm, 15 °C.

**Table 1:** Probes and target DNA oligonucleotides derived from influenza gene for neuraminidase.

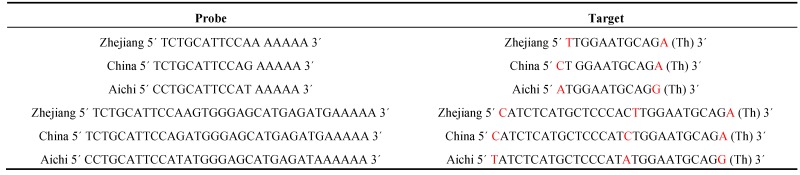

Red highlighted nucleotides represent a point mutation in the gene of H5N1 influenza for neuraminidase.

**Table 2:** Probes and target RNA oligonucleotides derived from influenza gene for neuraminidase.

Probe	Target
China 5´ UCUGCAUUCCAG AAAAA 3´	China 5´ CU GGAAUGCAGA (Th) 3´
China 5´ UCUGCAUUCCAGAUGGGAGCAUGAGAUGAAAAA 3´	China 5´ CAUCUCAUGCUCCCAUCUGGAAUGCAGA (Th) 3´

### 2.4. Characterization of ODN-QDs by MALDI-TOF/TOF

The ODN-SH-QDs were characterized using MALDI-TOF/TOF mass spectrometer Bruker Ultraflextreme (Bruker Daltonik GmbH, Bremen, Germany) equipped with a laser operating at wavelength of 355 nm with an accelerating voltage of 25 kV, cooled with nitrogen and a maximum energy of 50.4 µJ with repetition rate 2,000 Hz in reflector and positive mode, and with software for data acquisition and processing of mass spectra flexControl and flexAnalysis Version 3.4. 3‑hydroxypicolinic acid (3-HPA) was used as a matrix. The matrix 3-HPA was dissolved in ACS water to the concentration of 25 mg.mL^–1^ and 10 mg.mL^–1^ diammonium citrate (DAC) was added. Mixture was thoroughly vortexed for 2 min at room temperature. Working standard solutions were prepared daily by dilution of the stock solutions. The samples were prepared by dissolving with ACS water. 1 µL of matrix was applied on the floatchip target plate (Bruker) and dried under atmospheric pressure and room temperature. 1 µL oligo sample at a concentration of 40 µg.mL^–1^ was applied on top of this thin layer and dried under atmospheric pressure and room temperature. A mixture of oligo calibrations standard (15-mer, 20-mer and 25-mer) was used for calibration of the instrument.

### 2.5. Automatic Isolation of ODN-QDs

**Figure 1:**
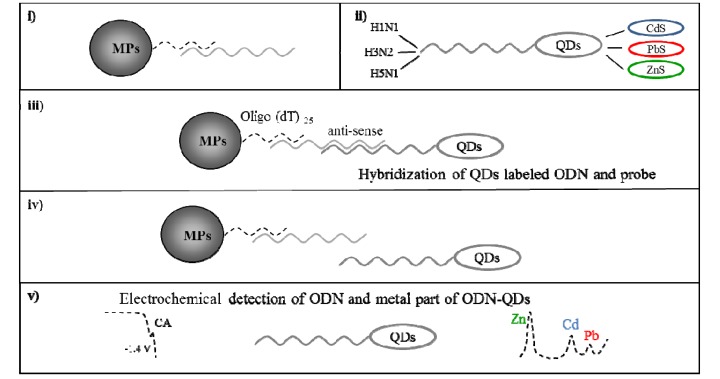
Scheme of fully automated paramagnetic particles (MPs)-based isolation followed by electrochemical detection of target ODN labeled with QDs. As a target, three different ODNs derived from point-mutated neuraminidase gene of influenza H5N1 were used. (**i**) Covalent binding between oligo (dT)_25_ and anti-sense sequence of different H5N1-derived ODNs; (**ii**) Target influenza ODNs labeled with QDs (China ODN with CdS, Aichi with PbS and Zhejiang with ZnS); (**iii**) Hybridization between anti-sense sequence bound to MPs and target sequence labeled with QDs; (**iv**) Isolation of target sequence ODN-QDs and elution from MPs (elution temperature 85 °C); (**v**) Electrochemical detection of ODN-QDs complex, ODN (CA peak) was measured by adsorptive transfer technique coupled with square wave voltammetry (AdTS SWV) and metal part of QDs (Cd, Pb and Zn peak) was measured by differential pulse anodic stripping voltammetry (DPASV).

Automatic pipetting station EP Motion 5075 (Eppendorf, Germany) with original devices (microplate holder, tips holder, tips (1,000, 300, 50 μL), rack tubes, reservoir holder, container for used tips, thermo adapter, PCR plate 96, magnetic adapter) was used for the fully automated target nucleic acids isolation process (Figure 1). Volume of 10 µL of Dynabeads® Oligo (dT)_25_ (Invitrogen, Oslo) was dispensed in selected wells in the plate (PCR 96, Eppendorf, Germany). Plate was subsequently transferred to the magnet and MPs storing solution was aspirated to waste. Subsequently, beads were further washed three times with 20 µL of phosphate buffer I (pH 6.5, 0.1 M NaCl + 0.05 M Na_2_HPO_4_ + 0.05 M NaH_2_PO_4_). The first hybridization was the next step. 10 µL of polyA-modified anti-sense oligonucleotides and 10 µL of hybridization buffer (0.1 M phosphate buffer, 0.6 M guanidinium thiocynate, 0.15 M Tris, pH 7.5) were added in each well and then the plate was incubated (15 min, 25 °C, mixing). This procedure was followed by a washing (three times) with 20 µL of phosphate buffer I. The second hybridization was the next step. 10 µL of QDs-labeled oligonucleotide and 10 µL of hybridization buffer (0.1 M phosphate buffer, 0.6 M guanidinium thiocynate, 0.15 M Tris, pH = 7.5) were added to selected wells and the plate was incubated (15 min, 25 °C, mixing). This procedure was followed by washing (three times) with 20 µL of phosphate buffer I. Then, 30 µL of elution solution (phosphate buffer II 0.2 M NaCl + 0.1 M Na_2_HPO_4_ + 0.1 M NaH_2_PO_4_) was added into selected wells and plate was subsequently incubated (5 min, 85 °C, mixing). After the elution step, the plate was transferred to the magnet, and the product from selected wells was transferred to separate wells after 120 s of elution step. This isolation assay was described previously [42,43,44,45].

### 2.6. Electrochemical Method for Detection of CA and Metal Peak of ODN-QDs

Electrochemical analyses were performed using a 663 VA Stand (Metrohm, Herisau, Switzerland) and a standard cell with three electrodes. It was equipped with a standard cell consisting of three electrodes, a cooled sample holder and a measurement cell set at 4 °C (Julabo F25, JulaboDE, Seelbach, Germany). The three-electrode system consisted of a hanging mercury drop electrode (HMDE) with a drop area of 0.4 mm^2^ as the working electrode, a Ag/AgCl/3M KCl reference electrode and a platinum electrode acting as the auxiliary. VA Database 2.2 by Metrohm was used for data acquisition and subsequent analysis. Acetate buffer (0.2 M CH_3_COOH + 0.2 M CH_3_COONa, pH 5.0) was used as a background electrolyte. Measurements were carried out at room temperature. The analyzed samples were deoxygenated prior to measurements by purging with argon (99.999%) saturated with water for 120 s. GPES 4.9 software was employed for data processing.

Cytosine-adenine reduction (CA peak) was detected by square wave voltammetry coupled with adsorptive transfer technique (AdTS SWV) [45,46,47,48,49,50]. The parameters of the electrochemical determination were as it follows: initial potential 0 V; end potential –1.85 V; frequency 80 Hz; potential step 0.005 V; amplitude 0.025 V. Time of accumulation was optimized.

Metal part of the ODN-QDs complex was detected by differential pulse anodic stripping voltammetry (DPASV). In this case the parameters of electrochemical determination were as it follows: Cd (initial potential –0.9 V; end potential –0.45 V, deposition potential –0.9 V); Zn (initial potential –1.2 V; end potential –0.85 V, deposition potential –1.2 V); Pb (initial potential –0.6 V; end potential –0.25 V, deposition potential –0.6 V); others parameters were same: equilibration time 5 s; modulation time 0.06; time interval 0.2 s; potential step 0.002 V; modulation amplitude 0.025. Time of accumulation was optimized.

### 2.7. Descriptive Statistics

Data were processed using MICROSOFT EXCELs (USA) and STATISTICA.CZ Version 8.0 (Czech Republic). The results are expressed as mean ±SD unless noted otherwise. Statistical significances of the differences were determined using STATISTICA.CZ. Differences with *p <* 0.05 were considered significant and were determined by using of one way ANOVA test (particularly Scheffe test), which was applied for means comparison.

## 3. Results and Discussion

Detection of a specific nucleic acid (NA) sequence is one of the most important markers of clinical medicine. Knowledge of a specific sequence of nucleic acid may be applied in modern therapeutic approaches [51,52]. Based on these facts, it is clear that there is a plenty of methods that have been suggested for NA analysis. Electrochemical methods are especially important due to low costs, rapidity, simplicity, and especially selectivity for specific NA sequences. Our system of isolation and detection requires less than one hour for one sample determination. Due this fact it is obvious that it is very quick way of determination opposite the other methods. Nanoparticles-based electrochemical NA analysis represents an important tool in the detection of specific NA sequences [53]. In addition, using nanoparticles provides us the possibility to use not only electrochemistry but also spectrometry. Moreover, nanoparticles have found application as oligonucleotide labels for electrochemical detection of viruses. Sun *et al.* used lead sulfide (PbS) nanoparticles as oligonucleotide labels for electrochemical detection of the 35 S promoter from cauliflower mosaic virus (CaMV) sequence [54]. Quantum dots (QDs)-labeled RNA oligonucleotide was used by Roh *et al.* for detection of hepatitis C virus nonstructural protein 5B (NS5B) [55]. In this study, magnetic particles with covalently boundoligooligo(dT)_25_ were used as a tool for isolation of complementary H5N1 chains (H5N1 Zhejin, China and Aichi, Figure 1). For detection of the isolated H5N1 chains, we used oligonucleotide chains of lengths of 12 (+5 adenine) or 28 (+5 adenine) bp labeled with quantum dots (CdS, ZnS and/or PbS, Figure 1). The scheme of the isolation and detection is shown in Figure 1. 

### 3.1. Characterization of DNA Oligonucleotide Labeled with QDs (CdS, PbS and ZnS) by MALDI-TOF/TOF

**Figure 2:**
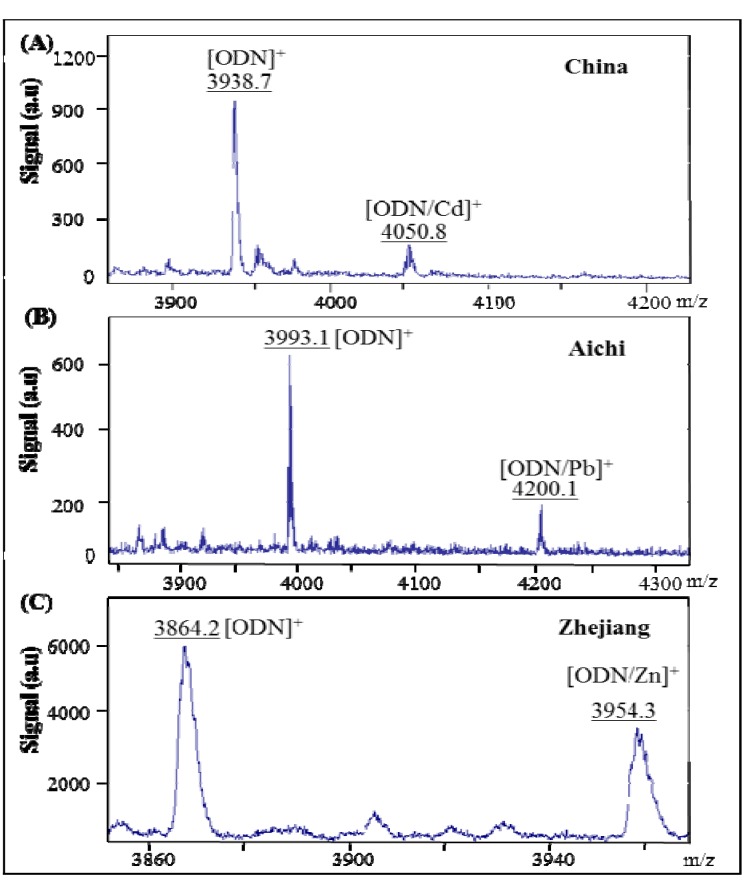
MALDI-TOF/TOF mass spectra of oligonucleotide (ODN) derived from H5N1 gene for neuraminidase labeled with QDs (CdS, PbS and ZnS). (**A**) Spectra of ODN and ODN labeled with CdS in China H5N1 sequence; (**B**) spectra of ODN and ODN labeled with PbS in Aichi H5N1 sequence; and (**C**) spectra of ODN and ODN labeled with ZnS in Zhejiang H5N1 influenza virus. 3-hydroxypicolinic acid (3-HPA) and diammonium citrate were used as matrix.

MALDI-TOF/TOF was used to characterize mass spectra of ODN-QDs in 3-hydroxypicolinic acid (3-HPA) and diammonium citrate as a matrix for testing the binding of H5N1 neuraminidase gene with quantum dots (CdS, ZnS and/or PbS). The spectra of each labeled ODN with different QDs are shown in Figure 2. The signals shown in Figure 2 were assigned as follows: (A) [ODN]^+^ of m/z 3,938.7 corresponding to unlabeled and [ODN/CdS]^+^ of m/z 4,050.8 corresponding to labeled complex with CdS in China H5N1 neuraminidase gene, (B) [ODN]^+^ of m/z 3,993.1 corresponding to unlabeled and [ODN/PbS]^+^ of m/z 4,200.1 corresponding to labeled complex with PbS in Aichi H5N1 neuraminidase gene and (C) [ODN]^+^ of m/z 3,864.2 corresponding to unlabeled and [ODN/ZnS]^+^ of m/z 3,954.3 corresponding to labeled with ZnS in Zhejiang H5N1 neuraminidase gene. It is evident that the suggested labeling of different ODNs with QDs was carried out correctly, as it is shown in Figure 1.

### 3.2. Scheme of Fully Automated MPs Isolation Followed by Electrochemical Detection of Target ODN Labeled with QDs

Nanoparticles have found their application in the rapid and simple isolation of nucleic acids from various biological matrixes. Magnetic nanoparticles represent an especially important improvement in these techniques due to very easy manipulation [56,57]. However, data about application of NPs in isolation of viral nucleic acid are very limited. Anti-sense oligonucleotides specific for conserved region of AIV PA protein of H5N1 avian influenza virus were used by Zhang *et al.* [58]. Nevertheless, the authors focused on the antiviral properties of these anti-sense oligonucleotides. In this study, we designed the protocol for multi-target isolation and detection of ODNs molecules (derived from neuraminidase gene of influenza H5N1) labeled with QDs. The first step consisted of hybridization (covalent binding) of the complementary anti-sense sequence modified with the polyA sequence on MPs modified with the polyT sequence (Figure 1i). This step was followed by influenza ODNs labeling with QDs. China ODNs (short and long) was labeled with CdS, Aichi with PbS and Zhejiang with ZnS (Figure 1ii) Hybridization between anti-sense and target ODN-QDs was the most important step (Figure 1iii). This step was the subject of study of effects of hybridization temperature and length of target ODN(s)-QDs on the hybridization, respectively the yield of isolated molecules. ODN-QDs elution was the last step of the isolation (temperature 85 °C, Figure 1iv). The isolation process was followed by electrochemical detection of ODN-QDs complex. Two voltammetry methods were used. ODN (CA peak) was determined by square wave voltammetry coupled with the adsorptive transfer technique (AdTS SWV) and metal peak (Cd, Pb and Zn) was determined by differential pulse anodic stripping voltammetry (DPASV), as it is shown in Figure 1v. Time of accumulation (t_A_) of both methods was optimized.

### 3.3. Optimization of AdTS SWV and DPASV Method

**Figure 3:**
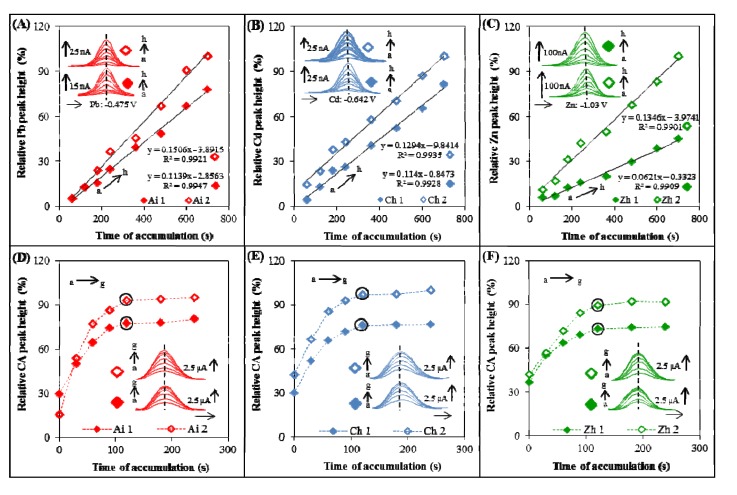
Optimization of electrochemical determination. The influence of time of accumulation on the heights of metal and CA peaks. (**A**–**C**) Dependence of relative height of metal peak (%) on the time of accumulation of ODN(s) labeled with QDs; (**D**–**E**) Dependence of relative height of CA peak (%) on the time of accumulation of ODN(s) labeled by QDs. In the optimization step, these ODN(s) labeled with QDs were used: (**A**+**D**) Aichi (Ai) was labeled with PbS (Ai 1 is short ODN–12 nucleotides, Ai 2 is long ODN–28 nucleotides), (**B**+**E**) China (Ch) was labeled with CdS (Ch 1 is short ODN–12 nucleotides, Ch 2 is long ODN–28 nucleotides), (**C**+**D**) Zhejiang (Zh) was labeled with ZnS (Zh 1 is short ODN–12 nucleotides, Zh 2 is long ODN–28 nucleotides). DPASV was used to determine metal, parameters were as it follows: initial potential –1.2 V (Zn), –0.8 V (Cd), –0.65 V (Pb); end potential –0.9 V (Zn), –0.5 V (Cd), –0.3 V (Pb), deposition potential –1.2 V (Zn), –0.8 V (Cd), –0.65 V (Pb); equilibration time 5 s; modulation time 0.06 s; time interval 0.2 s; potential step 0.002 V; modulation amplitude 0.025 V. Optimized parameter was time of accumulation (a → h): a 30 s, b 60 s, c 120 s, d 240 s, e 360 s, f 480 s, g 600 s, h 700 s. AdTS SWV method was used to determine CA peak, parameters were as it follows: purge time 60 s, initial potential –1.85 V; end potential 0 V; frequency 100 Hz; potential step 0.005 V; amplitude 0.025 V. Optimized parameter was time of accumulation (a → g): a 0 s, b 30 s, c 60 s, d 90 s, e 120 s, f 180 s, g 240 s.

Time of accumulation (t_A_) was optimized for detection of lead(II), cadmium(II) and zinc(II) ions using DPASV (Figure 3A–C, respectively). Different accumulation times (30, 60, 120, 240, 360, 480 and 600 s) were tested. Height of metal peak of ODN**-**QDs increased linearly up to 600 s, but the time of accumulation of 300 s was selected due to the time consuming measurements at t_A_ longer than 300 s. Time of accumulation (t_A_) was optimized also in the case of CA peak detection in various ODN-QDs complexes formed from Pb, Cd and Zn (Figure 3D–F, respectively). Tested time intervals were 0, 30, 60, 90, 120, 180 and 240 s. Time of accumulation 120 s was chosen as an optimal t_A_ as the highest peak at the lowest time of accumulation.

**Figure 4:**
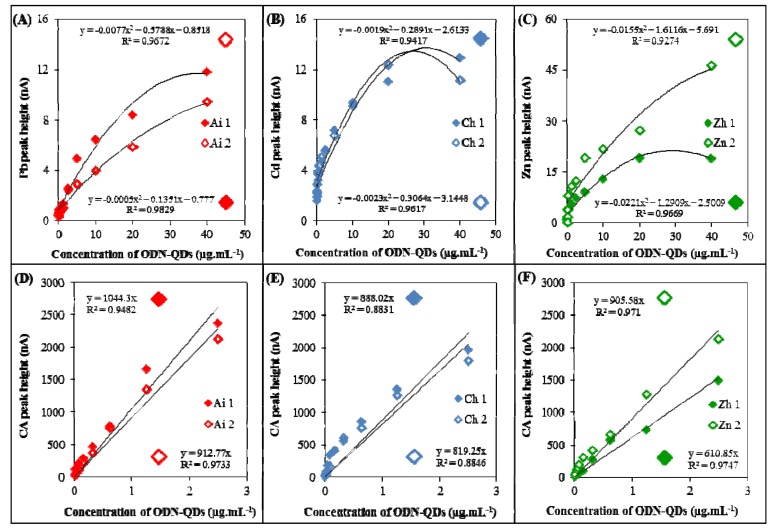
Calibration curves of metal and CA peaks. (**A**–**C**) Dependences of height of metal peak (nA) on concentration of ODN-QDs (µg/mL): (**A**) ODN Ai 1+2 (labeled with PbS), (**B**) Ch 1+2 (labeled with CdS), (**B**) Zh 1+2 (labeled with ZnS). Ai 1, Ch 1 and Zh 1 are 12 nucleotides long sequences labeled with QDs, Ai 2, Ch 2 and Zh 2 are 28 nucleotides long sequences labeled with QDs. To measure metal(s) in QDs, DPASV method was used (parameters are in caption in Figure 3); (**D**–**F**) Dependences of height of CA peak (nA) on concentration of ODN-QDs (µg/mL): (**D**) ODN Ai 1+2 (labeled with PbS), (**E**) Ch 1+2 (labeled with CdS), (**F**) Zh 1+2 (labeled with ZnS). AdTS SWV method was used for measurements (parameters are in caption in Figure 3).

Calibration of the metal peak (Pb, Cd and Zn) in ODN-QDs using DPASV was carried out and it is shown in Figure 4A–C, respectively. Ai ODN labeled with PbS demonstrated the highest coefficient of reliability followed with Zn and Cd. Further, we aimed at calibration of CA peak measured by AdTS SWV. CA peak was measured in various ODN-QDs complexes formed from Pb, Cd and Zn (Figure 4D–F, respectively). The highest coefficient of reliability was demonstrated by Zh ODN labeled with ZnS. 

### 3.4. Hybridization Experiments–Influence of Different Hybridization Condition of Hybridization Efficiency

#### 3.4.1. Influence of Temperature and Different Length of ODN-QDs on Hybridization

**Figure 5:**
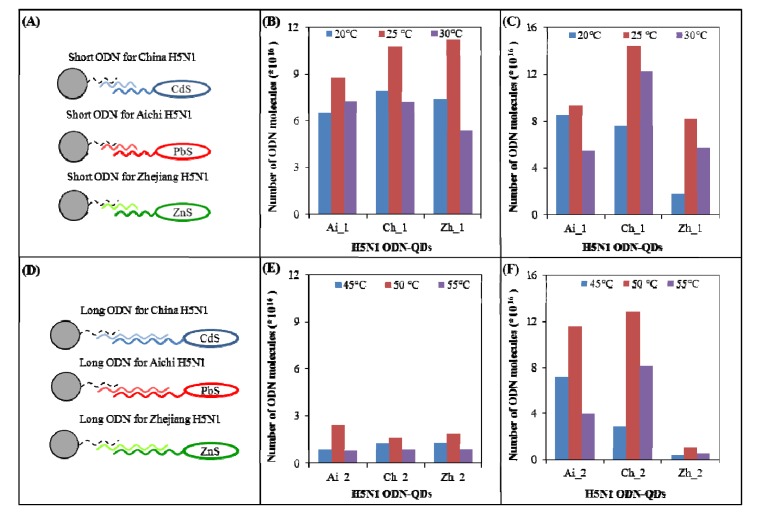
The effect of different temperatures on hybridization of short and long ODN-QDs derived from H5N1 gene for neuraminidase. (**A**) Short and/or (**D**) long ODN anti-sense (anti-H5N1 Zhejiang, China and Aichi) modified MPs with covalently bound oligo(dT)_25_ were used as a tool for isolation of complementary H5N1 strands (H5N1 Zhejin, China and Aichi) labeled with QDs (CdS, ZnS and/or PbS). (**B**+**C**) The effect of the temperature of hybridization (20, 25 and 30 °C) on number of hybridized molecules of short ODN-QDs abbreviated as Ai 1, Ch 1 and Zh 1; (**B**) Number of H5N1 molecules was calculated from the height of CA peak; (**C**) Number of H5N1 molecules was calculated from the height of metal peak. (**E**+**F**) The effect of the temperature of hybridization (45 °C, 50 °C and 55 °C) on number of hybridized molecules of long ODN-QDs abbreviated as Ai 2, Ch 2 and Zh 2; (**E**) Number of H5N1 molecules was calculated from the height of CA peak; (**F**) Number of H5N1 molecules was calculated from the height of metal peak. AdTS SWV method was used to measure the height of CA peak was used (parameters are in caption in Figure 3). DPASV method was used to measure the height of metal peak (parameters are in caption in Figure 3). Blue color: minimal temperature of hybridization was 20 °C for 12 nucleotides long ODNs and/or 45 °C for 28 nucleotides long ODNs, red color: optimal temperature of hybridization was 25 °C for 12 nucleotides long ODNs and/or 50 °C for 28 nucleotides long ODNs, violet color: minimal temperature of hybridization was 30 °C for 12 nucleotides long ODNs and/or 55 °C for 28 nucleotides long ODNs.

Firstly, we investigated the effect of length of ODN and temperature of hybridization on an amount of isolated ODN-QDS (effectiveness of hybridization) in accordance with the scheme introduced in Figure 5A,D, where hybridization of short (12 nucleotides + 5A tail; Ai 1, Ch 1 a Zh 1) and long (28 nucleotides + 5A tail; Ai 2, Ch 2 a Zh 2) nucleotide sequences is shown, respectively. Double hybridization represents a substantial part of isolation. The first step was based on the hybridization of anti-sense chain of MPs (oligo (dT)_25_), and the second one consisted in the second hybridization between anti-sense chain and target ODN-QDs molecule (Aichi 1, 2 labeled by PbS; China 1, 2 labeled by CdS a Zhejiang 1, 2 labeled by ZnS).

Effectiveness of the hybridization of short nucleotide sequences was monitored at different temperatures: 20 °C, 25 °C and 30 °C. Effectiveness of the hybridization of long nucleotide sequences was monitored at 45 °C, 50 °C and 55 °C. These temperatures were suggested in accordance with a calculation of *T_m_* for given length and sequence of nucleotide. For short nucleotide sequences (12 nucleotides + 5A tail), the Tm value was *T_m_ = (wA+xT) × 2 + (yG + zC) × 4*, for long nucleotide sequences the TM value was *T_m_ = 64.9 +41 × (yG + zC-16.4)/(wA + xT + yG + zC)*, where w, x, y, and z are the numbers of the bases A, T, G, and C in the sequence of ODN, respectively. Temperature of hybridization (*T_hyb_*) was calculated according to T_hyb_ = T_m_–10 °C (hybridization temperature specific to the probe is typically 5 °C–10 °C low than T_m_). Subsequently, hybridization minimal, optimal and maximal values were suggested. Number of the isolated molecules was determined indirectly according to the height of peak that was measured using DPASV and AdTS SWV. CA peak that corresponds to the signals of cytosine and adenine was measured using AdTS SWV (Figure 5B,E) and metal peak was measured by DPASV (Figure 5C,F).

Effectiveness of the hybridization of short nucleotide sequences. To detect the highest CA and metal peaks, temperature of hybridization of 25 °C was selected as the most suitable for the short nucleotide sequences. Effectiveness of hybridization calculated according to the height of CA peak between three different ODN-QDs (Ai1, Ch1, Zh1) showed no significant differences (Figure 5B). Effectiveness of hybridization calculated according to the height of metal peak showed significant differences at *p* < 0.05 (the most effective Ch1-labeled CdS and the least effective Zh1-labeled ZnS), as it is shown in Figure 5C. 

Effectiveness of the hybridization of long nucleotide sequences. According to CA and metal peaks heights, the temperature of hybridization of 50 °C was chosen as the most suitable for the hybridization of long nucleotide sequences. Effectiveness of hybridization calculated according the height of CA peak between three different ODN-QDs (Ai2, Ch2, Zh2) is not comparable to short ODN (Figure 5E). The most effective isolation was determined in the case of Ai 2 (labeled by PbS), on the other hand the least effective in the case of Ch2 and Zh2 that were labeled with CdS (Ch2) and ZnS (Zh2), respectively (Figure 5F). According to the number of the isolated molecules calculated from the height of metal peak, the most effective ODN were Ai2 and Ch2 and the least effective Zh2.

In conclusion, the largest number of short ODN-QDS molecules was isolated at 25 °C and the largest number of long ODN-QDS molecules was isolated at 50 °C. These temperatures were used in the subsequent experiment, where effectiveness of hybridization of mixture of short and long ODN-QDs was investigated.

#### 3.4.2. Effect of Different Lengths of Mixture of Short and Long ODN-QDS on Hybridization

**Figure 6:**
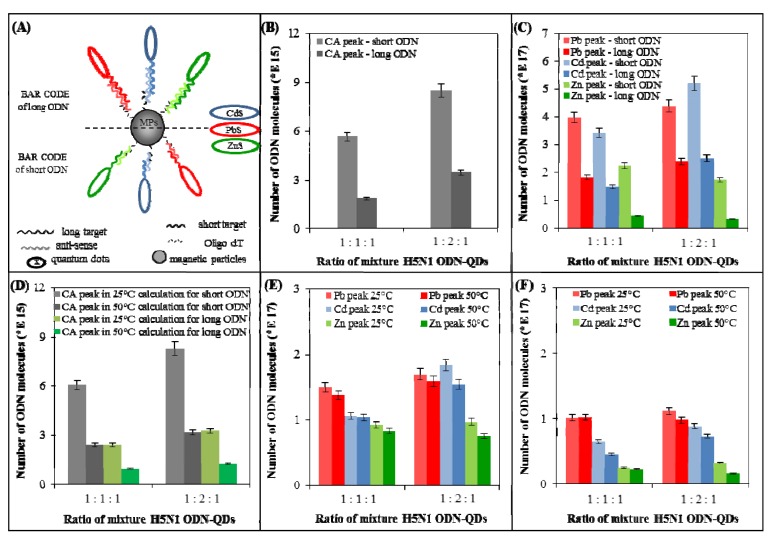
The effect of optimal temperature on hybridization of short, long and mixture of short and long ODN-QDs derived from H5N1 gene for neuraminidase. (**A**) Experimental scheme: bar code array for detection of three different (long and/or short) point-mutated H5N1 neuraminidase gene. (**B**+**C**) The effect of optimal temperature on hybridization (optimal temperature: 25 °C for mixture of short ODN-QDs and 50 °C for mixture of long ODN-QDs) on number of hybridized short ODN-QDs molecules. Two ratios of oligonucleotides (1:1:1–the same ratio of oligonucleotides, 1:2:1 oligonucleotides labeled with CdS were in twofold ratio than ODN labeled with ZnS and PbS; (**B**) The effectiveness of hybridization determined by the height of CA peak. Number of molecules calculated from the height of CA peak (light grey–CA peak of short ODN, dark grey–CA peak of long ODN); (**C**) The effectiveness of hybridization was monitored using the height of Cd, Pb and Zn peaks. Number of molecules calculated from the height of metal peak (light color–metal peak(s) of short ODN, dark color–metal peak(s) of long ODN; (**D**–**F**) The effect of optimal temperature of hybridization (optimal temperature: 25 °C in light color and 50 °C in dark color, mixture of short and long ODN-QDs) on mixture of short and long ODN-QDs (12+28 nucleotides) molecules. Two ratios of oligonucleotides (1:1:1–the same ratio of oligonucleotides, 1:2:1 oligonucleotides labeled with CdS were in twofold ratio than that labeled with ZnS and PbS) were investigated; (**D**) The effectiveness of hybridization determined using the height of CA peak. (**E**+**F**) The number of molecules calculated from the height of metal peak (light color—metal peak of short ODN, dark color–metal peak of long ODN); (**E**) The effectiveness of hybridization was calculated from short ODN-QDs; (**F**) effectiveness of hybridization was calculated from long ODN-QDs.

Hybridization of both mixtures was carried out according to the scheme introduced in Figure 6A. This experiment focused on the comparison of effectiveness of hybridization of mixture of ODN-ODs with short and long nucleotide sequences. Calculation of number of isolated molecules was carried out according to formula for Ai 1 (short ODN-QDs) and Ai 2 (long ODN-QDs). Mixtures of ODN-QDs were prepared at different rates of all three components as 1:1:1–the same ratio of oligonucleotides, and 1:2:1 oligonucleotides labeled with CdS were in twofold ratio than that labeled with ZnS and PbS. This was done due to evaluation of the suggested procedure, because we expected the increase of peaks corresponding to CdS labeled ODNs.

Comparison of results of effectiveness of hybridization of mixture of short and long ODN-QDs is shown in Figure 6B,C. The effect of hybridization was calculated based on the number of the isolated molecules calculated from the height of CA peak (Figure 6B) and from the height of metal peak (Figure 6C), where resulting values of the isolation of mixture of short molecules (lighter color) and mixture of long molecules (darker color) is shown. We monitored the effect of length in the mixture of short and long ODN-QDs as well as different ratios of individual components of ODN-QDs mixture. More effective is hybridization of the mixture of short ODN due to the better and rapid hybridization. In addition, they probably exhibit lower specificity. We proved also the effect of the ratio of different components in ODN-QDs mixture on the hybridization. Ratio 1:2:1 brought larger number of the isolated molecules compared to ratio 1:1:1. Similarly, we monitored the effect of the length of nucleotide sequence and their different ratios on number of the isolated molecules. More effective hybridization (larger number of isolated molecules) is observable for short ODN-QDs.

#### 3.4.3. Influence of Two Optimal Hybridization Temperatures (25 °C and 50 °C) on Efficiency of Hybridization of Mixture of Short and Long ODN-QDs

Figure 6D–F introduces the effect of two temperatures of hybridization (25 °C and 50 °C) on the hybridization of mixture containing all ODN-QDs (Ai1+2, Ch 1+2, Zh 1+2). The number of isolated molecules was calculated from the height of CA peak (Figure 6D) and from the height of metal peak (Figure 6E,F). For the calculating of number of the isolated ODNs, formulas for Ai 1 (Figure 6D, E, grey columns) and for Ai 2 (Figure 6D, F, green columns) were used. Light color columns indicate results of hybridization at 25 °C, dark color columns at 50 °C. We tested two different ratios of mixture of short and long ODN-QDs. Ratio 1:1:1 represents mixture of all short and long ODN-QDs, where individual components occur at the same rate (columns on the left). Ratio 1:2:1 represents mixture of all short and long ODN-QDs, where ODN labeled with CdS (Ch1 and Ch2) occur at double rate compared to that labeled with PbS and ZnS (columns on the right).

Figure 6D shows effectiveness of hybridization (number of isolated molecules) at 25 °C that is larger compared to 50 °C. This is caused by the fact that short ODNs are not able of hybridization at 50 °C, because their T_m_ is almost for 20 °C lower. On the other hand, long ODN-QDs hybridized only at 50 °C. In conclusion, short sequences were hybridized at 25 °C specifically and long sequences nonspecifically, thus, the yield of the isolated molecules was higher at 25 °C compared to 50 °C. Calculation of number of isolated molecules was carried out according to the formula for short ODN-QDs (Figure 6D, grey columns) or for long ODN-QDs (Figure 6D, green columns). Columns on the right indicate increase in number of the isolated molecules. This fact is based on the increased ratio of ODNs labeled with CdS (Ch1 and Ch2).

Figure 6E shows results of hybridization calculated from the height of metal peak. Formula for Ai 1 was used for the calculation of effectiveness of hybridization. Differences between light and dark columns (hybridization at 25 °C and 50 °C) are not so evident in comparison with those that are shown in Figure 6D, where the yield was calculated from the height of CA peak. At 25 °C both short and long ODNs are hybridized, whereas at 50 °C long ODNs giving smaller signal are hybridized only. Method for detection of individual metals is not influenced by this effect. Significant difference is well evident in the increase in Cd peak in column with the ratio of 1:2:1 (*p* < 0.05).

Figure 6F shows results of hybridization calculated from the height of metal peak. However, the formula for Ai 2 was used for calculation compared to Figure 6E. Differences between light and dark columns (hybridization at 25 °C and 50 °C) are not so evident compared to Figure 6D, where the yield was calculated from the height of CA peak. In addition, the increase in the number of isolated molecules is well evident also in the case of columns calculated from Pb peak at the ratio of 1:1:1. Increase in the Cd peak in column with the ratio of 1:2:1 is well evident compared to ratio of 1:1:1; otherwise the values of isolated molecules between two different ratios are similar.

#### 3.4.4. Isolation and Detection of RNA ODN-QDs

Above mentioned results showed that suggested procedure allowed to distinguish individual point mutations and therefore could be applicable for detecting of the point mutation in real samples. Influenza viruses belong to the RNA viruses group and therefore we were interested in the issue whether we would be able to detect RNA sequence as a model of the real sample using the suggested procedure. The detection of RNA sequences was performed under the optimal conditions (determined for DNA sequences). Application of the same experimental conditions for DNA and RNA sequences is in accordance with numerous published papers [59,60,61,62,63,64,65,66,67,68,69,70]. The RNA ODN are much more sensitive to external conditions and optimization of detection is usually provided with DNA ODN (DNA analogues), also in the case of RNA pathogens [59,60,61,62,63,64,65,66,67,68,69,70].

**Figure 7:**
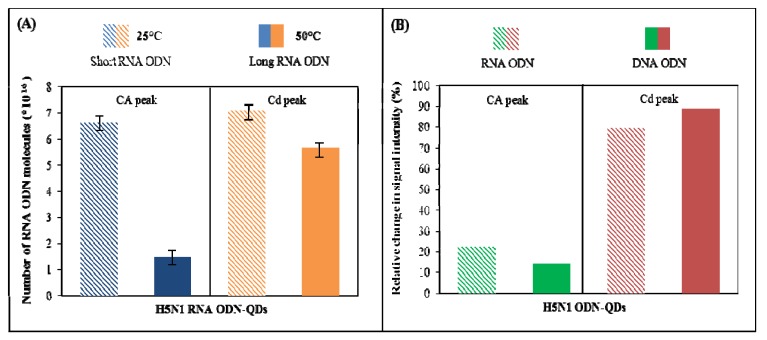
(**A**) The effect of the RNA-ODN length on the number of hybridized molecules. Two long varieties of China RNA ODN sequences were tested due to CA and Cd peak detection. Short RNA ODN sequence was hybridized at 25 °C and long RNA ODN sequence was hybridized at 50 °C; (**B**) Percentage of signal intensity of the long ODN sequence related to the short ODN sequence detected due to CA and Cd peak for RNA and DNA ODN sequence.

For our purposes, we decided to use two length China RNA ODN-CdS to confirm that the method is applicable to analysis of real sample. In accordance with above experimental design, one short (12 nucleotides) and one long (28 nucleotides) RNA ODN-CdS were tested. The differences between DNA and RNA experimental design were in number of isolated sequences (DNA: three different short and three different long ODN in combination with three different QDs and on the other hand RNA: one short, one long RNA sequence and one QDs type. The RNA ODNs were labeled with CdS quantum dots as in the case of DNA ODNs. The comparison of various detection ways is shown in Figure 7A. This picture shows number of RNA ODN molecules due to the detection via CA and Cd peaks. The comparison for short and long RNA ODNs is presented too. It is evident that the detection of short RNA ODN led to the same results compared to DNA ODNs. Analyzing long RNA ODNs showed various results, because Cd peak gave higher number of target molecules. This is in accordance with the results shown in Figure 5 for DNA ODNs. The changes of detected signals caused by the changing length of ODNs were compared too. The similarity assessment of these between RNA and DNA ODNs is shown in Figure 7B. There is the influence of RNA or DNA ODNs on detected signals quantified. Here is the percentage of signal intensity of the long ODN sequence related to the short ODN sequence detected due to CA and Cd peak. It is obvious that the compared signals reach the similar values with differences to ten percentages. 

Presented results also show that the application of RNA ODNs to the suggested procedure brings no issues and behavior of the RNA ODNs and DNA analogues is similar. Due these facts, one may suggest that the optimized procedure is applicable for real sample analysis. 

## 4. Conclusions

Mutations of the influenza virus in combination with the global increasing of the use of anti‑influenza specific drugs allows for the selection of antiviral drug-resistant viruses. Therefore, there is imperative to have faster and more sensitive assays for detection of antiviral drug-resistant strains of the influenza virus. New assays are important for the surveillance of HPAI H5N1 and for decisions to use specific antiviral drugs in the treatment of human patients suspected or suffering from H5N1 influenza. In this study, we demonstrate methods for multi-target detection of NAIs-resistant influenza subtypes. This multi target bar code assay is based on the electrochemical analysis of isolated target molecules labeled with QDs. Based on previously published papers [42,43], we designed and optimized hybridization assay for multi-target isolation and detection of influenza H5N1 neuraminidase gene, respectively point-mutated neuraminidase gene. These point mutations are responsible for the resistance to NAIs. Revealing NAIs’ resistance is important for targeted and successful treatment of severe human influenza cases, and for preventing the occurrence of severe influenza disease, especially HPAI H5N1.

## References

[1] Shoham D. (2013). Influenza type A virus: An outstandingly protean pathogen and a potent modular weapon. Crit. Rev. Microbiol..

[2] Plourde J.R., Pyles J.A., Layton R.C., Vaughan S.E., Tipper J.L., Harrod K.S. (2012). Neurovirulence of H5N1 infection in ferrets is mediated by multifocal replication in distinct permissive neuronal cell regions. PLoS One.

[3] Gilbert M., Jambal L., Karesh W.B., Fine A., Shiilegdamba E., Dulam P., Sodnomdarjaa R., Ganzorig K., Batchuluun D., Tseveenmyadag N. (2012). Highly pathogenic avian influenza virus among wild birds in mongolia. PLoS One.

[4] Wei K.F., Chen Y.F., Chen J., Wu L.J., Xie D.X. (2012). Evolution and adaptation of hemagglutinin gene of human H5N1 influenza virus. Virus Genes.

[5] Abdelwhab E.M., Hafez H.M. (2012). Insight into alternative approaches for control of avian influenza in poultry, with emphasis on highly pathogenic H5N1. Viruses.

[6] Zhang J.F. (2012). Advances and future challenges in recombinant adenoviral vectored H5N1 influenza vaccines. Viruses.

[7] Weinheimer V.K., Becher A., Tonnies M., Holland G., Knepper J., Bauer T.T., Schneider P., Neudecker J., Ruckert J.C., Szymanski K. (2012). Influenza A viruses target type II pneumocytes in the human lung. J. Infect. Dis..

[8] Chen F., Yan Z.Q., Liu J., Ji J., Chang S., Liu D., Qin J.P., Ma J.Y., Bi Y.Z., Xie Q.M. (2012). Phylogenetic analysis of hemagglutinin genes of 40 H9N2 subtype avian influenza viruses isolated from poultry in China from 2010 to 2011. Virus Genes.

[9] Zhao J.Q., Wang X., Ragupathy V., Zhang P.H., Tang W., Ye Z.P., Eichelberger M., Hewlett I. (2012). Rapid detection and differentiation of swine-origin influenza A virus (H1N1/2009) from other seasonal influenza A viruses. Viruses.

[10] Alberts B. (2012). INTRODUCTION H5N1. Science.

[11] Takekawa J.Y., Prosser D.J., Newman S.H., Bin Muzaffar S., Hill N.J., Yan B.P., Xiao X.M., Lei F.M., Li T.X., Schwarzbach S.E. (2010). Victims and vectors: Highly pathogenic avian influenza H5N1 and the ecology of wild birds. Avian Biol. Res..

[12] Leung Y.H.C., Cheung P., Zhang L.J., Wu Y.O., Chow K.C., Ho C.K., Chow C.K., Ng C.F., Li B., Tsang C.L. (2011). Influenza viruses in wild birds in Hong Kong, 2003–2010. Influenza Other Respir. Viruses.

[13] Capua I., Alexander D.J. (2008). Ecology, epidemiology and human health implications of avian influenza viruses: Why do we need to share genetic data?. Zoonoses Public Health.

[14] Neumann G., Chen H., Gao G.F., Shu Y.L., Kawaoka Y. (2010). H5N1 influenza viruses: Outbreaks and biological properties. Cell Res..

[15] Bragstad K., Jorgensen P.H., Handberg K., Hammer A.S., Kabell S., Fomsgaard A. (2007). First introduction of highly pathogenic H5NI avian influenza A viruses in wild and domestic birds in Denmark, Northern Europe. Virol. J..

[16] Rebel J.M.J., Peeters B., Fijten H., Post J., Cornelissen J., Vervelde L. (2011). Highly pathogenic or low pathogenic avian influenza virus subtype H7N1 infection in chicken lungs: Small differences in general acute responses. Vet. Res..

[17] Comin A., Klinkenberg D., Marangon S., Toffan A., Stegeman A. (2011). Transmission dynamics of low pathogenicity avian influenza infections in Turkey flocks. PLoS One.

[18] Kabir S.M.L. (2010). Avian flu (H5N1): Threat of “global pandemic” is growing and it’s impact on the developing countries’ economy. Afr. J. Microbiol. Res..

[19] Yamada S., Suzuki Y., Suzuki T., Le M.Q., Nidom C.A., Sakai-Tagawa Y., Muramoto Y., Ito M., Kiso M., Horimoto T. (2006). Haemagglutinin mutations responsible for the binding of H5N1 influenza A viruses to human-type receptors. Nature.

[20] Fauci A.S., Collins F.S. (2012). Benefits and risks of influenza research: lessons learned. Science.

[21] Nguyen T., Rivailler P., Davis C.T., Hoa D.T., Balish A., Dang N.H., Jones J., Vui D.T., Simpson N., Huong N.T. (2012). Evolution of highly pathogenic avian influenza (H5N1) virus populations in Vietnam between 2007 and 2010. Virology.

[22] Tang D.J., Lam Y.M., Siu Y.L., Lam C.H., Chu S.L., Peiris J.S.M., Buchy P., Nal B., Bruzzone R. (2012). A single residue substitution in the receptor-binding domain of H5N1 hemagglutinin is critical for packaging into pseudotyped lentiviral particles. PLoS One.

[23] Park A.W., Glass K. (2007). Dynamic patterns of avian and human influenza in east and southeast Asia. Lancet Infect. Dis..

[24] Rao S.S., Styles D., Kong W., Andrews C., Gorres J.P., Nabel G.J. (2009). A gene-based avian influenza vaccine in poultry. Poult. Sci..

[25] Webster R.G., Govorkova E.A. (2006). Focus on research: H5N1 influenza–Continuing evolution and spread. N. Engl. J. Med..

[26] Peiris J.S.M., de Jong M.D., Guan Y. (2007). Avian influenza virus (H5N1): A threat to human health. Clin. Microbiol. Rev..

[27] Uyeki T.M., Bresee J.S. (2007). Detecting human-to-human transmission of avian influenza a (H5N1). Emerg. Infect. Dis..

[28] Hayden F.G., de Jong M.D. (2011). Emerging influenza antiviral resistance threats. J. Infect. Dis..

[29] Cai Z.P., Ducatez M.F., Yang J.L., Zhang T., Long L.P., Boon A.C., Webby R.J., Wan X.F. (2012). Identifying antigenicity-associated sites in highly pathogenic H5N1 influenza virus hemagglutinin by using sparse learning. J. Mol. Biol..

[30] Zhao G., Zhong L., Lu X.L., Hu J., Gu X.B., Kai Y., Song Q.Q., Sun Q., Liu J.B., Peng D.X. (2012). Characterisation of a highly pathogenic H5N1 clade 2.3.2 influenza virus isolated from swans in Shanghai, China. Virus Genes.

[31] Nidom C.A., Yamada S., Nidom R.V., Rahmawati K., Alamudi M.Y., Kholik, Indrasari S., Hayati R.S., Horimoto K.I., Kawaoka Y. (2012). Genetic characterization of H5N1 influenza viruses isolated from chickens in Indonesia in 2010. Virus Genes.

[32] Huang K., Zhu H.V., Fan X.H., Wang J., Cheung C.L., Duan L., Hong W.S., Liu Y.M., Li L.F., Smith D.K. (2012). Establishment and lineage replacement of H6 influenza viruses in domestic ducks in Southern China. J. Virol..

[33] Miyoshi-Akiyama T., Akasaka Y., Oogane T., Kondo Y., Matsushita T., Funatogawa K., Kirikae T. (2012). Development and evaluation of a line probe assay for rapid typing of influenza viruses and detection of the H274Y mutation. J. Virol. Methods.

[34] Redlberger-Fritz M., Aberle S.W., Strassl R., Popow-Kraupp T. (2012). Rapid identification of neuraminidase inhibitor resistance mutations in seasonal influenza virus A(H1N1), A(H1N1)2009, and A(H3N2) subtypes by melting point analysis. Eur. J. Clin. Microbiol. Infect. Dis..

[35] Bao J.R., Huard T.K., Piscitelli A.E., Tummala P.R., Aleemi V.E., Coon S.L., Master R.N., Lewinski M.A., Clark R.B. (2011). Reverse-transcription polymerase chain reaction/pyrosequencing to characterize neuraminidase H275 residue of influenza A 2009 H1N1 virus for rapid and specific detection of the viral oseltamivir resistance marker in a clinical laboratory. Diagn. Microbiol. Infect. Dis..

[36] Deng Y.M., Caldwell N., Hurt A., Shaw T., Kelso A., Chidlow G., Williams S., Smith D., Barr I. (2011). A comparison of pyrosequencing and neuraminidase inhibition assays for the detection of oseltamivir-resistant pandemic influenza A(H1N1) 2009 viruses. Antivir. Res..

[37] Leang S.K., Deng Y.M., Shaw R., Caldwell N., Iannello P., Komadina N., Buchy P., Chittaganpitch M., Dwyer D.E., Fagan P. (2013). Influenza antiviral resistance in the Asia-Pacific region during 2011. Antivir. Res..

[38] Chairat K., Tarning J., White N.J., Lindegardh N. (2013). Pharmacokinetic properties of anti-influenza neuraminidase inhibitors. J. Clin. Pharmacol..

[39] Li H., Shih W.Y., Shih W.H. (2007). Synthesis and characterization of aqueous carboxyl-capped CdS quantum dots for bioapplications. Ind. Eng. Chem. Res..

[40] Hennequin B., Turyanska L., Ben T., Beltran A.M., Molina S.I., Li M., Mann S., Patane A., Thomas N.R. (2008). Aqueous near-infrared fluorescent composites based on apoferritin-encapsulated PbS quantum dots. Adv. Mater..

[41] Genbank. http://www.ncbi.nlm.nih.gov/genbank/.

[42] Krejcova L., Huska D., Hynek D., Kopel P., Adam V., Hubalek J., Trnkova L., Kizek R.  (2013). Using of paramagnetic microparticles and quantum dots for isolation and electrochemical detection of influenza viruses’ specific nucleic acids. Int. J. Electrochem. Sci..

[43] Krejcova L., Hynek D., Kopel P., Adam V., Hubalek J., Trnkova L., Kizek R. (2013). Paramagnetic particles isolation of influenza oligonucleotide labelled with CdS QDs. Chromatographia.

[44] Huska D., Adam V., Babula P., Trnkova L., Hubalek J., Zehnalek J., Havel L., Kizek R. (2011). Microfluidic robotic device coupled with electrochemical sensor field for handling of paramagnetic micro-particles as a tool for determination of plant mRNA. Microchim. Acta.

[45] Huska D., Hubalek J., Adam V., Vajtr D., Horna A., Trnkova L., Havel L., Kizek R. (2009). Automated nucleic acids isolation using paramagnetic microparticles coupled with electrochemical detection. Talanta.

[46] Krejcova L., Dospivova D., Ryvolova M., Kopel P., Hynek D., Krizkova S., Hubalek J., Adam V., Kizek R. (2012). Paramagnetic particles coupled with an automated flow injection analysis as a tool for influenza viral protein detection. Electrophoresis.

[47] Zitka O., Krizkova S., Krejcova L., Hynek D., Gumulec J., Masarik M., Sochor J., Adam V., Hubalek J., Trnkova L. (2011). Microfluidic tool based on the antibody-modified paramagnetic particles for detection of 8-hydroxy-2'-deoxyguanosine in urine of prostate cancer patients. Electrophoresis.

[48] Prasek J., Huska D., Jasek O., Zajickova L., Trnkova L., Adam V., Kizek R., Hubalek J. (2011). Carbon composite micro- and nano-tubes based electrodes for detection of nucleic acids. Nanoscale Res. Lett..

[49] Huska D., Zitka O., Krystofova O., Adam V., Babula P., Zehnalek J., Bartusek K., Beklova M., Havel L., Kizek R. (2010). Effects of cadmium(II) ions on early somatic embryos of Norway spruce studied by using electrochemical techniques and nuclear magnetic resonance. Int. J. Electrochem. Sci..

[50] Chomoucka J., Drbohlavova J., Masarik M., Ryvolova M., Huska D., Prasek J., Horna A., Trnkova L., Provaznik I., Adam V. (2012). Nanotechnologies for society. New designs and applications of nanosensors and nanobiosensors in medicine and environmental analysis. Int. J. Nanotechnol..

[51] Berton M., Turelli P., Trono D., Stein C.A., Allemann E., Gurny R. (2001). Inhibition of HIV-1 in cell culture by oligonucleotide-loaded nanoparticles. Pharm. Res..

[52] Schneider T., Becker A., Ringe K., Reinhold A., Firsching R., Sabel B.A. (2008). Brain tumor therapy by combined vaccination and antisense oligonucleotide delivery with nanoparticles. J. Neuroimmunol..

[53] Cai H., Zhu N.N., Jiang Y., He P.G., Fang Y.Z. (2003). Cu@Au alloy nanoparticle as oligonucleotides labels for electrochemical stripping detection of DNA hybridization. Biosens. Bioelectron..

[54] Sun W., Zhong J.H., Qin P., Jiao K. (2008). Electrochemical biosensor for the detection of cauliflower mosaic virus 35 S gene sequences using lead sulfide nanoparticles as oligonucleotide labels. Anal. Biochem..

[55] Roh C., Lee H.Y., Kim S.E., Jo S.K. (2010). A highly sensitive and selective viral protein detection method based on RNA oligonucleotide nanoparticle. Int. J. Nanomed..

[56] Bandyopadhyay A., Chatterjee S., Sarkar K. (2011). Rapid isolation of genomic DNA from E. coli XL1 Blue strain approaching bare magnetic nanoparticles. Curr. Sci..

[57] Trachtova S., Kaman O., Spanova A., Veverka P., Pollert E., Rittich B. (2011). Silica-coated La0.75Sr0.25MnO3 nanoparticles for magnetically driven DNA isolation. J. Sep. Sci..

[58] Zhang T., Zhao P.S., Zhang W., Liang M., Gao Y.W., Yang S.T., Wang T.C., Qin C., Wang C.Y., Xia X.Z. (2011). Antisense oligonucleotide inhibits avian influenza virus H5N1 replication by single chain antibody delivery system. Vaccine.

[59] Malecka K., Grabowska I., Radecki J., Stachyra A., Gora-Sochacka A., Sirko A., Radecka H. (2012). Voltammetric detection of a specific DNA sequence of avian influenza virus H5N1 using HS-ssDNA probe deposited onto gold electrode. Electroanalysis.

[60] Ganbold E.O., Kang T., Lee K., Lee S.Y., Joo S.W. (2012). Aggregation effects of gold nanoparticles for single-base mismatch detection in influenza A (H1N1) DNA sequences using fluorescence and Raman measurements. Colloid Surf. B-Biointerfaces.

[61] Liu X.G., Cheng Z.Q., Fan H., Ai S.Y., Han R.X. (2011). Electrochemical detection of avian influenza virus H5N1 gene sequence using a DNA aptamer immobilized onto a hybrid nanomaterial-modified electrode. Electrochim. Acta.

[62] Lai W.A., Lin C.H., Yang Y.S., Lu M.S.C. (2012). Ultrasensitive and label-free detection of pathogenic avian influenza DNA by using CMOS impedimetric sensors. Biosens. Bioelectron..

[63] Tian J.P., Zhao H.M., Liu M., Chen Y.Q., Quan X. (2012). Detection of influenza A virus based on fluorescence resonance energy transfer from quantum dots to carbon nanotubes. Anal. Chim. Acta.

[64] Chung D.J., Kim K.C., Choi S.H. (2011). Electrochemical DNA biosensor based on avidin-biotin conjugation for influenza virus (type A) detection. Appl. Surf. Sci..

[65] Fan H., Ju P., Ai S.Y. (2010). Controllable synthesis of CdSe nanostructures with tunable morphology and their application in DNA biosensor of Avian Influenza Virus. Sens. Actuator B-Chem..

[66] Adam V., Huska D., Hubalek J., Kizek R. (2010). Easy to use and rapid isolation and detection of a viral nucleic acid by using paramagnetic microparticles and carbon nanotubes-based screen-printed electrodes. Microfluid. Nanofluid..

[67] Chen X.J., Xie H., Seow Z.Y., Gao Z.Q. (2010). An ultrasensitive DNA biosensor based on enzyme-catalyzed deposition of cupric hexacyanoferrate nanoparticles. Biosens. Bioelectron..

[68] Lim S.H., Buchy P., Mardy S., Kang M.S., Yu A.D.C. (2010). Specific nucleic acid detection using photophysical properties of quantum dot probes. Anal. Chem..

[69] Tam P.D., Hieu V.N., Chien N.D., Le A.T., Tuan M.A. (2009). DNA sensor development based on multi-wall carbon nanotubes for label-free influenza virus (type A) detection. J. Immunol. Methods.

[70] Kim S.A., Kim S.J., Lee S.H., Park T.H., Byun K.M., Kim S.G., Shuler M.L. (2009). Detection of avian influenza-DNA hybridization using wavelength-scanning surface plasmon resonance biosensor. J. Opt. Soc. Korea.

